# The Microtubule Inhibitor Podofilox Inhibits an Early Entry Step of Human Cytomegalovirus

**DOI:** 10.3390/v8100295

**Published:** 2016-10-24

**Authors:** Tobias Cohen, Toni M. Schwarz, Frederic Vigant, Thomas J. Gardner, Rosmel E. Hernandez, Benhur Lee, Domenico Tortorella

**Affiliations:** Department of Microbiology, Icahn School of Medicine at Mount Sinai, New York, NY 10029, USA; tobias.cohen@icahn.mssm.edu (T.C.); toni.schwarz@mssm.edu (T.M.S.); frederic.vigant@mssm.edu (F.V.); Thomas.gardner@icahn.mssm.edu (T.J.G.); rosmel.hernandez@icahn.mssm.edu (R.E.H.); benhur.lee@mssm.edu (B.L.)

**Keywords:** cytomegalovirus, microtubules, virus entry, podofilox, colchicine, nocodazole

## Abstract

Human cytomegalovirus is a ubiquitous β-herpesvirus that infects many different cell types through an initial binding to cell surface receptors followed by a fusion event at the cell membrane or endocytic vesicle. A recent high-throughput screen to identify compounds that block a step prior to viral gene expression identified podofilox as a potent and nontoxic inhibitor. Time-of-addition studies in combination with quantitative-PCR analysis demonstrated that podofilox limits an early step of virus entry at the cell surface. Podofilox was also able to drastically reduce infection by herpes simplex 1, an α-herpesvirus with a very similar entry process to CMV. Podofilox caused a reduced maximal plateau inhibition of infection by viruses with single step binding processes prior to fusion-like Newcastle disease virus, Sendai virus, and influenza A virus or viruses that enter via endocytosis like vesicular stomatitis virus and a clinical-like strain of CMV. These results indicate that microtubules appear to be participating in the post-binding step of virus entry including the pre- and post-penetration events. Modulation of the plasma membrane is required to promote virus entry for herpesviruses, and that podofilox, unlike colchicine or nocodazole, is able to preferentially target microtubule networks at the plasma membrane.

## 1. Introduction

Cytomegalovirus (CMV) is a complex β-herpesvirus expressing over 250 genes capable of manipulating multiple host cell processes. The virus is transmitted to HCMV negative hosts either vertically, from mother to the developing fetus, or horizontally through bodily secretions including saliva and breast milk. While infections in immunocompetent adults and children are generally asymptomatic or subclinical, CMV establishes a permanent, latent infection capable of reactivating at a later time [1]. In the immunocompromised and neonatal populations, CMV is a major source of morbidity and mortality [2]. CMV is the leading cause of permanent congenital disability in the United States [3] and also a source of organ transplant failure [4,5,6].

CMV therapeutics are severely lacking, not only in terms of the number of treatment options, but also due to the severe toxicity of the currently available pharmaceuticals. The majority of anti-CMV therapeutics target the viral DNA polymerase, and while intended to be specific for the viral polymerase, they often target the host polymerase as well, causing pancytopenia and gastrointestinal distress. Currently, there are no effective therapeutics approved for use in pregnant women to protect against congenital CMV infection. Additionally, many of the approved drugs generate drug resistant virus mutants [7,8,9,10,11,12]. Consequently, there is a dire need for new CMV therapies, and the first step to creating them is to identify novel drug targets by better understanding the viral lifecycle within the host. In this article, we propose a new role for cellular microtubules in CMV entry that may be targeted by future therapeutics.

CMV entry involves the viral envelope proteins gM and gN that bind to heparan sulfate proteoglycans on the cell surface [13,14]. Following this initial attachment, the viral glycoprotein gB enhances viral attachment to the cell by binding to β1 integrins [15,16]. The virus then enters the cell by one of two pathways, depending on not only the strain of the virus but also the cell type from which the virus originated [17]. In the lab adapted AD169 strain, the virus lacks the glycoprotein complex containing UL131a and thus enters the cell via fusion at the plasma membrane [18,19]. In clinical-like strains such as TB40/E, the UL128/130/-131a complex is present, and rather than penetrate at the cell membrane, the virus is endocytosed and fuses with the endocytic vesicle [20,21]. Regardless of whether the virus fuses with the cell membrane or endocytic membrane, the viral capsid then translocates through the cytosol to the nucleus along microtubules [22]. Disruption of the microtubule network with nocodazole was reported to prevent the viral capsid from reaching the nucleus, but the virus was still able to enter the cell and subsequently resume translocation when the microtubule network was restored [23]. The seminal paper demonstrating this relied solely on nocodazole treatment to disrupt the cellular microtubule network, however, not all microtubule inhibitors act in the same manner, nor do all microtubules react the same to various microtubule inhibitors. Tubulin polymerizing inhibitors such as nocodazole, colchicine, and podofilox all have different structures but bind to overlapping sites on the tubulin monomer, and they competitively inhibit one another [24]. This, in combination with the fact that the microtubule networks within the cell do not behave uniformly [25], led us to believe that microtubule polymerization inhibitors other than nocodazole might inhibit CMV through a different mechanism. To that effect, we characterized the mode of action of podofilox and colchicine, compounds identified in a high-content screen [26] as effective inhibitors of CMV infection. In this paper, we demonstrate that in contrast to colchicine, podofilox, a podophyllotoxin-derived microtubule polymerization inhibitor, blocks a post-binding event during the initial steps of viral entry into fibroblasts.

## 2. Materials and Methods

### 2.1. Cell Lines, Antibodies, Viruses and Chemicals

Human lung fibroblasts (MRC5) (ATCC (American Type Culture Collection), Manassas, VA, USA) were cultured in complete Dulbecco’s modified Eagle’s medium (DMEM) supplemented with 10% fetal bovine serum, 100 U of penicillin-streptomycin/mL and 100 U of HEPES/mL, at 37 °C in a humidified incubator with 5% CO_2_. Human retinal pigmented epithelial cells (ARPE-19) (ATCC, Manassas, VA, USA) were cultured in a 1:1 mix of Dulbecco’s modified Eagle’s medium and Kaighn’s modification of Ham’s F-12 medium supplemented with 10% fetal bovine serum, 100 U of penicillin-streptomycin/mL and 100 U of HEPES/mL, at 37 °C in a humidified incubator with 5% CO_2_. Human cytomegalovirus AD169_WT_ was purchased from ATCC (Cat. No. ATCC VR538) as a genetically unmodified virus. AD169_WT_, AD169_IE2-YFP_, TB40/E_WT_, and TB40/E_FLAG-YFP_ were propagated as previously described [27,28]. AD169_IE2-YFP_ (gift from Dr. Leor Weinberger (Gladstone Institute, University of San Francisco, San Francisco, CA USA) was generated by inserting EYFP (Mountain View, CA USA) to the 3′ end of IE2 exon 5 in the parent pAD/Cre AD169 bacterial artificial chromosome (BAC) clone [29,30,31]. The TB40/E_WT_ lacking any genetic modifications and TB40/E Bac4 DNA clone [32] used to generate TB40/E_FLAG-YFP_ [28] were kind gifts from Dr. Christian Sinzger (University of Ulm, Ulm, Germany). Infectious virus yield was assayed on MRC5 cells by median tissue culture infective dose (TCID_50_). The TCID_50_ results were used to estimate infectious particles/mL to infect cells at the desired multiplicity of infection (MOI). Influenza A virus encoding *Gaussia* luciferase (IFV^Luc^) was provided by the lab of Dr. Peter Palese [33]. Vesicular stomatitis virus (VSV)-GFP, herpes simplex 1 (HSV1)-GFP, Newcastle disease virus (NDV)-GFP, Sendai virus (SeV)-GFP and the broad-spectrum antiviral JL122 were used as previously described [34,35,36]. Podofilox, nocodazole and colchicine were purchased from Sigma-Aldrich (St. Louis, MO, USA).

### 2.2. Time-of-Addition Experiments

MRC5 cells (1.0 × 10^4^ in 100 μL) were plated in a 96-well plate (Greiner, Kremsmünster, Austria). The following day media was replaced with 95 µL of DMEM. Compound (5 µL of 20× stock) was added to the wells at the designated time points relative to virus infection (range −1 h p.i. to 2 h p.i.) in sextuplicate. The final concentrations were chosen to inhibit virus by more than 50%. Cells were infected at 0 h p.i. with AD169_IE2-YFP_ (MOI 3), and at 18 h p.i. the plates were analyzed with an Acumen ^e^X3 cytometer (TTP Labtech, Cambridge, MA, USA) for the number of IE2-YFP positive cells/well based on IE2-YFP fluorescent intensity/well [27]. Using DMSO pretreated cells infected with AD169_IE2-YFP_ as 100% infection, the percent infection of cells treated with drug at different time points relative to infection was determined.

### 2.3. Virus Entry Assays

Three separate experiments to address CMV entry were performed. (1) MRC5 cells (2.5 × 10^5^ in 2 mL) were plated in a 6-well plate. The following day the cells were pretreated with drugs for 1 h and MRC5 cells were infected for 2 h on ice with AD169_WT_ (MOI 3). Cells were then washed with PBS and removed by cell scraper; (2) MRC5 cells (2.5 × 10^5^ in 2 mL) were plated in a 6 well plate (Greiner, Kremsmünster, Austria). The following day cells were pretreated with 50 nM podofilox, 500 nM colchicine, or 5 µM nocodazole for 1 h and MRC5 cells were infected for 2 h with wild type AD169 (AD169_WT_) (MOI 3). Cells were washed with 3× with PBS, incubated with trypsin to remove non-penetrated virus from the cells, and the DNA was extracted from cells using the QIAGEN mini DNA extraction kit (Qiagen Sciences, Germantown, MD, USA). qPCR was performed using SYBR green analyzed on a Roche LightCycler 480 (Roche, Basel, Switzerland) with primers targeting human β-actin and CMV unique long (UL)123 (β-actin forward primer: 5′-CATTGCCGACGGATGCA-3′, β-actin reverse primer: 5′-GCCGATCCACACGGAGTACT-3′, UL123 forward primer: 5′-GCCTTCCCTAAGACCACCAA-3′, UL123 reverse primer: 5′-ATTTTCTGGGCATAAGCCATAATC-3′). The amount of viral DNA in each sample relative to β-actin was calculated and viral DNA was expressed as % virus bound or internalized using DMSO-treated samples as 100%. (3) MRC5 cells (2.5 × 10^5^ in 2 mL) were plated in a 6 well plate. The following day the cells were pretreated with drugs for 1 h and MRC5 cells were infected for 2 h on ice with AD169_WT_ (MOI 3). Cells were then washed with PBS and removed by cell scraper to retain bound, non-entered virus, and their DNA extracted and quantified.

### 2.4. Penetration Assay

MRC5 cells (1.0 × 10^4^ in 100 μL) were plated in a 96-well plate. The following day, the medium was replaced with 100 μL of DMEM containing 500 nM, 50 nM, or 5 nM of Podofilox or 0.01% DMSO for 1 h prior to infection with AD169_IE2-YFP_ (MOI 3). Cells were placed at 4 °C for 1 h to allow for viral attachment then washed with citrate buffer pH 3.0 or pH 7.0 or incubated at 37 °C for 1 h as previously described [37,38]. At 18 h p.i., the plates were analyzed with an Acumen ×3 cytometer for the number of IE2-YFP positive cells/well based on IE2-YFP fluorescent intensity/well. The % infection was determined using DMSO treated cells as 100%.

### 2.5. Plaque Reduction Assay

MRC5 cells were seeded in triplicate with DMEM at a density of 5 × 10^4^ cells/well in a 24-well plate. The next day, cells were pretreated 1 h with: 0.1% DMSO; 12 μM ganciclovir; 0.5, 5, 50, and 500 nM of podofilox, colchicine, or nocodozole. Following AD169_IE2-YFP_ infection (MOI 0.1) for 2 h with the indicated drugs, the cells were washed twice with DMEM and incubated with media containing 10 μg/mL of Cytogam (CSL Behring, King of Prussia, PA, USA). At 5 d p.i., cells were fixed in 4% PFA and stained with Giemsa (Harleco, EMD Millipore, Bellerica, MA, USA). Wells were washed with dH_2_O and plaques were counted using phase contrast microscopy.

### 2.6. Reversibility Assay

MRC5 cells (1.0 × 10^4^ in 100 μL) were plated in a 96-well plate. The following day the medium was replaced with 100 µL of 50 nM podofilox at various times prior to infection for 2 h, so that each sextuplicate column would have progressively longer periods of time elapse between the cessation of podofilox treatment and infection (range of 0−8 h elapsed between treatment cessation and infection). Cells were then infected with AD169_IE2-YFP_ (MOI 3) and at 18 h p.i., the plates were analyzed with an Acumen ^e^X3 cytometer for the number of IE2-YFP positive cells/well based on IE2-YFP fluorescent intensity/well [27]. Using cells treated with DMSO immediately prior to infection (0 h elapsed between treatment cessation and infection) with AD169_IE2-YFP_ as 100% infection, the percent infection of cells treated with drug at different times prior to infection was determined.

### 2.7. Determining Half Maximal Effective Concentration (EC_50_) of Podofilox on the Expression of IE Genes by the Clinical Strain TB40/E

ARPE-19 cells (1.0 × 10^4^ in 100 μL) were plated in a 96-well plate. The following day, the medium was replaced with DMEM:modified F12 media (see ARPE-19 culture conditions) with varying concentrations (0–5 μM) of the compounds (final volume of 100 μL) in sextuplicate for 1 h at 37 °C and then cells were infected with AD169_IE2-YFP_ (MOI 3) or TB40/E_WT_ (MOI 3). At 18 h p.i., cells were fixed in 4% PFA and incubated at 4 degrees with an anti-IE1 antibody conjugated with Alexa488 (Mab810, Millipore Co., Bellerica, MA, USA) for 1 h. Cells were then analyzed with an Acumen ^e^X3 cytometer for fluorescent intensity from Alexa488. Fluorescent emission above 2 standard deviations of background was registered as positive signal. Any fluorescent signal larger than 5 μm smaller than 300 μm, and separated from any other emission by at least 0.5 μm in both x and y axes was classified as an “event”. Using DMSO treated cells infected with TB40/E_WT_ as 100% infection, the percent infection of cells pre-treated with increasing amounts of compound was determined. The EC_50_ values were calculated using GraphPad PRISM 5’s nonlinear fit log (inhibitor) vs. response (three parameters) analysis of the average % infection values [26].

### 2.8. Analysis of Podofilox to Inhibit Diverse Viruses

MRC5 cells (1.0 × 10^4^ in 100 μL) were plated in a 96-well plate. The following day the medium was replaced with DMEM with varying concentrations (0–5 μM) of podofilox (final volume 100 μL) for 1 h at 37 °C before the cells were infected with influenza virus (IAV)^Luc^, VSV-GFP, HSV1-GFP, NDV-GFP, or SeV-GFP. At 8 h p.i. (VSV) and 24 h p.i. (HSV1, NDV, SeV), the plates were analyzed with an Acumen ^e^X3 cytometer for the number of GFP positive cells/well based on GFP fluorescent intensity/well. The IFV^Luc^ infection was analyzed at 24 h p.i. for *Gaussia* luciferase activity using the Cytation^3^ imaging reader (BioTek, Winooski, VT, USA) as described in [33]. The EC_50_ values were determined as described above.

### 2.9. Analysis of Cells Treated with Microtubule Inhibitors by Microscopy

Glass coverslips were placed in a 6-well plate and MRC5 cells (2.0 × 10^5^ in 2 mL) were placed in each well. The following day, medium was replaced with 50 nM podofilox, 500 nM colchicine, or 5 μM nocodazole for 1 h prior to fixation with 4% paraformaldehyde for 10 mins. Cells were then treated for 30 min in blocking solution and stained for 1 h with murine anti-tubulin antibody. Cells were then stained with Hoescht and lapine anti-mouse antibody conjugated to an Alexa 488 fluorophore. Glass coverslips were removed from wells and placed on slides. Cells were analyzed (60×) using the Evos FL cell imaging system.

### 2.10. Statistical Analysis

All statistical analysis of results performed in this manuscript were done in Microsoft excel using a two tailed student’s T-test of means. Statistical significance was determined as *p* < 0.05, with *p* < 0.01 and *p* < 0.001 designated as well.

## 3. Results

### 3.1. Podofilox Blocks an Early Step of CMV Entry

A high-content screen to discover compounds that target the early steps of virus infection identified the three classes of microtubule inhibitor including podofilox, colchicine, and vincrisitine [26] that block a CMV infection.

**Figure 1:**
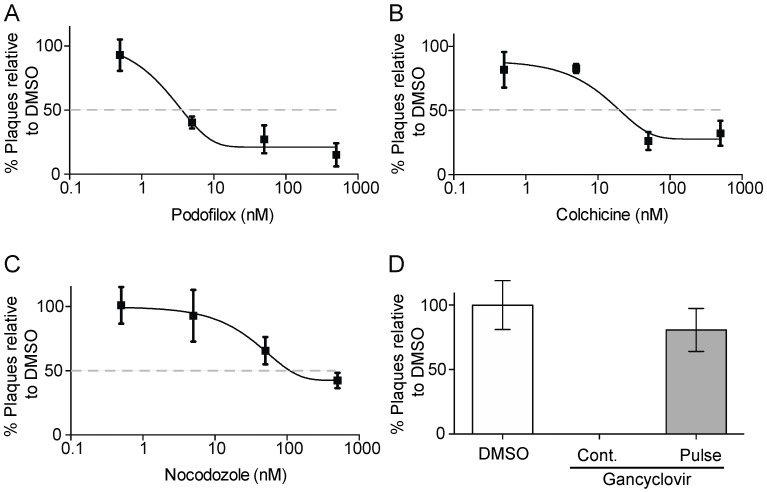
Microtubule inhibitors limit viral dissemination. MRC5 cells pre-treated with 0.5, 5, 50, and 500 nM of podofilox (**A**); colchicine (**B**); nocodazole (**C**) or ganciclovir (12 μM) (**D**, Pulse) were infected with AD169IE2-YFP (MOI 0.1) for 2 h. The cells were washed and analyzed for plaques 7 d p.i. As a control, cells were continuously (Cont.) treated with ganciclovir (12 μM) (**D**). The % Plaques was determined using DMSO-treated cells as 100%. The error bars represent the standard deviation from six replicates.

Interestingly, subsequent studies found that only podofilox and colchicine were effective at inhibiting a CMV infection with low EC50 values of 30 nM and 180 nM, respectively, and CC50 values >5000 nM [26]. To demonstrate the efficacy of microtubule inhibitors to limit viral proliferation, a plaque reduction assay was performed using podofilox and colchicine as well as nocodazole, a previously characterized microtubule inhibitor [23] (Figure 1). MRC5 cells were pre-treated podofilox, colchicine, nocodozole (0.5, 5, 50, and 500 nM) or gancyclovir (12 μM) followed up an AD169_IE2-YFP_ infection (MOI 0.1). Subsequently, the drug was removed to prevent drug cytoxicity and analyzed for viral plaques 7 days post infection (d p.i.). Podofilox, colchicine, and nocolozole inhibited viral plaques with EC50 values of 8 nM, 50 nM, and 100 nM, respectively (Figure 1A–C). As a control, cells treated with gancyclovir (12 μM) for 7 days inhibited the generation of plaques (Figure 1D). However, the microtubule inhibitors were toxic to cells upon continous treatment for 7 days (data not shown). Strikingly, the data demonstrate that the microtubule inhibtors were effective at decreasing plaque numbers. The EC50 values of podofilox and colchicine in the plaque reduction assay were slightly lower than the EC50s from the infection assay [26] probably due to the sensitive of the infection assay. Collectively, the microtubule inhibitors effectively inhibited virus infection and subsequent dissemination.

**Figure 2:**
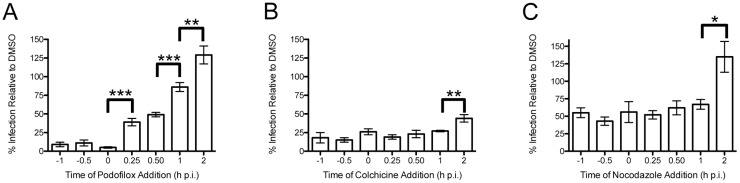
Anti-CMV pharmacokinetics is varied among microtubule inhibitors. MRC5 cells were infected with AD169_IE2-YFP_ and treated with 50 nM podofilox (**A**); 500 nM colchicine (**B**); and 5 μM nocodazole (**C**) at various times relative to infection. Infected cells were analyzed for fluorescent intensity at 18 h p.i. to determine the number of virus-infected cells. The percent infection was normalized to cells receiving DMSO pretreatment as 100%. Columns represent mean of six replicates and error bars show standard error. * *p* < 0.05, ** *p* < 0.01, *** *p* < 0.001.

To further characterize the mode of action of podofilox and colchicine, we performed time-of-addition studies to define which step of CMV infection is affected by these inhibitors and how this compares to MRC5 cells infected with the AD169_IE2-YFP_ reporter virus (MOI 3) were treated with podofilox, colchicine, or nocodazole at concentrations capable of inhibiting CMV infection by >50% from 1 h prior (−1) to 2 h post infection (h p.i.) (Figure 2). These values are well below the CC50 values previously determined [23]. The analysis of AD169_IE2-YFP_-infected cells using an Acumen ^e^X3 cytometer for a robust fluorescent signal in the nucleus ~18 h p.i. [27] found that podofilox, colchicine and nocodazole treatment significantly reduced virus infection (10%–20% and ~50% relative infection, respectively) when added, prior to (−1 and −0.5 h p.i.) and 0 h p.i. (Figure 2). Interestingly, podofilox treatment was most effective as a pretreatment because the addition of podofilox 0.25 h p.i. reduced its efficacy by over 4-fold (from 9% to 39% relative infection) and had limited efficacy when added at 1 h p.i. (86% relative infection) (Figure 2A). In contrast, colchicine and nocodazole treatment maintained full efficacy as an antiviral when added up to 1 h p.i. (27% and 67% relative infection, respectively) only showing a decrease in efficacy when added 2 h p.i. (Figure 2B,C). Interestingly, colchicine still partially inhibited CMV infection (44% infection relative to DMSO) at 2 h p.i., while nocodazole treatment was completely ineffective at the same time point post-infection. Collectively, podofilox targets a different step during the initial phase of infection than colchicine or nocodazole. Remarkably, podofilox was effective during the earliest time points of virus infection that includes the binding, pre-penetration, and post-penetration steps of virus entry. Thus, the mode of action of podofilox will be pursued because its mechanism of action is unique compared to the other microtubule inhibitors.

### 3.2. Podofiox Inhibits a Post-Binding CMV Entry Step

**Figure 3:**
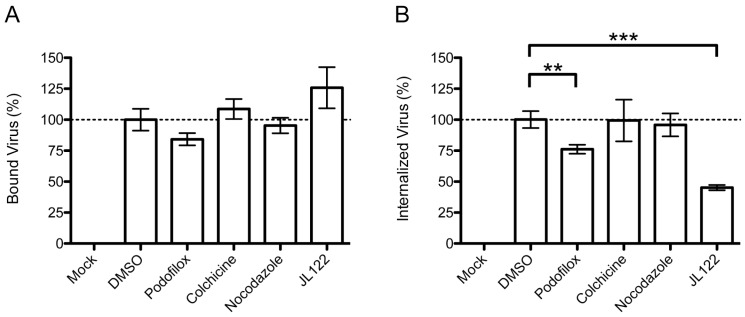
Podofilox inhibits CMV entry post binding. MRC5 cells or virus were pretreated with DMSO, podofilox (50 nM), colchicine (500 nM), nocodazole (5 μM), and JL122 (250 nM) for 1 h (or virus was treated for 1 h in the case of JL122) and infected with AD169_WT_ for 2 h at either 4 °C (**A**) or 37 °C (**B**) and analyzed for the presence of viral DNA by qPCR; (**A**) Bound Virus (%) and (**B**) Internalized Virus (%) values were determined using the relative number of virus genomes (see Materials and Methods) from DMSO treated cells as 100%. Graph represents the average of three separate experiments each performed in triplicate, error bars correspond to standard error. ** *p* < 0.01, *** *p* < 0.001.

To determine which step of virus entry was inhibited by podofilox, we performed a series of experiments that would assay either virus attachment to the cellular surface or penetration of the virus across the cellular membrane and into the cytoplasmic space. First, we quantified viral genomes from virions that have attached to the cellular surface (Figure 3A). MRC5 cells pretreated with DMSO, podofilox, colchicine, or nocodazole at concentrations that effectively limit infection and incubated for one hour at 37 **°**C then infected with AD169_WT_ (MOI 3) on ice for 2 h to prevent viral penetration. Cells were harvested using a cell scraper so that the bound virus would be measured by qPCR analysis. The % bound virus was determined using relative number of virus genome from DMSO-treated cells as 100% (Figure 3A). None of the treatments resulted in a substantial reduction in CMV DNA suggesting that none of the compounds were able to inhibit the binding of CMV to MRC5 cells.

Next, an entry assay quantified intracellular viral genomes from virions that penetrated the cellular membrane in the presence of drug treatment. MRC5 cells were pre-treated with the indicated drugs for one hour at 37 **°**C then infected with AD169_WT_ (MOI 3) for 2 h at 37 **°**C (Figure 3B). Cells were harvested by trypsinization so that any bound, un-entered virus would be removed prior to qPCR analysis. The relative amount of viral genomes was referred to as % internalized virus and determined using DMSO-treated cells as 100% (Figure 3B). As a control, cells were infected with virus pretreated with JL122. The fusion inhibitor JL122 is a light activated compound that oxidizes unsaturated phospholipids within the highly curved virus membrane, preventing viral membrane fusion with the host cell [27,28,39,40]. Strikingly, podofilox significantly reduced the amount of viral DNA from cells treated at 37 **°**C by 24% (*p* < 0.01) (Figure 3B). As expected, JL122-treatment significantly reduced the DNA viral load by 55% (Figure 3A) due to its function as a viral fusion inhibitor. On the other hand, colchicine and nocodazole did not affect viral entry into cells, consistent with a previous finding that nocodazole does not prevent CMV entry [23]. Together, the results support the model that podofilox targets a step during viral penetration into the cell.

**Figure 4:**
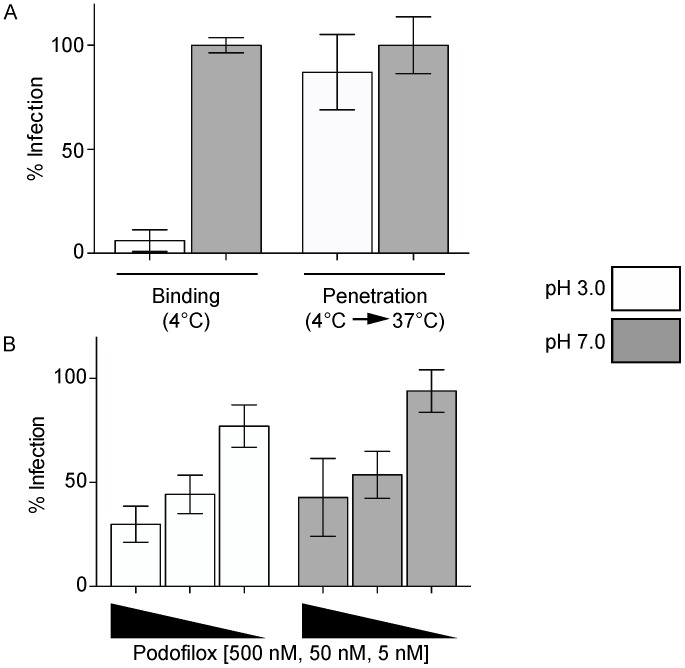
Podofilox blocks a latter step during CMV entry. (**A**) MRC5 cells infected with AD169_IE2-YFP_ (MOI 3) at 4 °C were either washed with either a pH 3.0 buffer (white bars) or pH 7.0 buffer (gray bars) (Binding) or shifted to 37 °C for 1 h and then washed with a pH 3.0 or pH 7.0 buffer (Penetration); (**B**) MRC5 cells pretreated with podofilox were infected with AD169_IE2-YFP_ (MOI 3) at 4 °C, shifted to 37 °C for 1 h and then washed with a pH 3.0 or pH 7.0 buffer. For both (**A**) and (**B**); virus infection was analyzed an Acumen X3 cytometer (18 h p.i.) and the % infection was determined using DMSO treated pH 7.0 washed cells as 100% infection. The error bars correspond to standard error of six replicates.

To further explore which step of virus entry is affected by podofilox treatment, a penetration experiment was performed using low pH to remove and inactivate non-entered virus [38] (Figure 4). To demonstrate the effectiveness of a low pH was to remove non-entered virus, MRC5 cells infected with AD169_IE2-YFP_ at 4 °C were washed with a pH 3.0 or pH 7.0 buffer and were analyzed (18 h p.i.) for virus infection using an Acumen X3 cytometer (Figure 4A, Binding). Under these conditions, the low pH wash dramatically reduced virus infection by inactivating virus. However, shifting the cells to 37 °C for 1 h to allow for virus penetration followed by a low pH wash did not affect virus infection when compared to the neutral pH wash (Figure 4A, Penetration). Note, a low pH wash after a 15 min incubation at 37 °C inactivated >95% of the virus (data not shown). These findings suggest that low pH is effective at removing weakly bound virus and inactivating unbound virus.

To determine if podofilox targets virus penetration, MRC5 cells pretreated with podofilox (0.005, 0.05, and 0.5 μM) were infected with AD169_IE2-YFP_ (MOI 3) at 4 °C, shifted to 37 °C for 1 h, washed with a pH 3.0 or pH 7.0 buffer and then analyzed for virus-infected cells 18 h p.i. (Figure 4B). Interestingly, there is a similar level of virus inhibition in a concentration dependent manner of podofilox-treated infected cells washed with low or neutral pH. Thus, the results suggest that podofilox is blocking a step immediately down-stream of virus penetration. Alternatively, virus entry is a multi-step process that includes a pre-penetration step in which virus attachment to the cell via multiple cellular proteins that would be insensitive to a low pH wash. This model would be consistent with Figure 3B that a decrease of viral genome was observed from podofilox-treated cells following trypsinization. Collectively, the data supports the model that podofilox limits virus entry by targeting a post-binding step.

**Figure 5:**
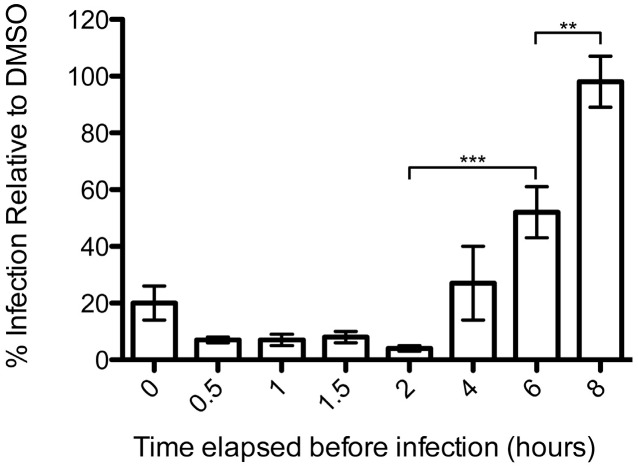
Podofilox’s effect on CMV infection is reversible. MRC5 cells pretreated with 50 nM podofilox were infected with AD169_IE2-YFP_ and analyzed for fluorescent intensity at 18 h p.i. to determine the number of virus-infected cells. The percentage infection was normalized to cells receiving DMSO pretreatment as 100%. Columns represent average of six replicates and error bars correspond to standard error. ** *p* < 0.01, *** *p* < 0.001.

To further characterize podofilox’s mechanism of action, the reversibility of podofilox treatment of virus-infected cells was examined (Figure 5). MRC5 cells pre-treated (2 h) with podofilox had drug removed for up to 8 h prior to infection with AD169_IE2-YFP_ (MOI 3). Virus infection was quantified at ~18 h p.i. with an Acumen ^e^X3 cytometer and IE2-YFP positive cells was used to determine % infection. Podofilox treatment was quite effective for at least 2 h post-treatment and was rescued beginning at 4 h post-treatment and completely restored at 8 h post-treatment. These findings imply that podofilox’s effect on microtubules may be due to its non-covalent inhibition [24] or upregulated expression dynamics of tubulin in response to short term/low dose treatment of microtubule depolymerizing agents [41]. Thus, the inhibition of CMV infection by podofilox can be rescued shortly after cessation of treatment.

### 3.3. Efficacy of Podofilox to Inhibit a Clinical-Like CMV Strain

**Figure 6:**
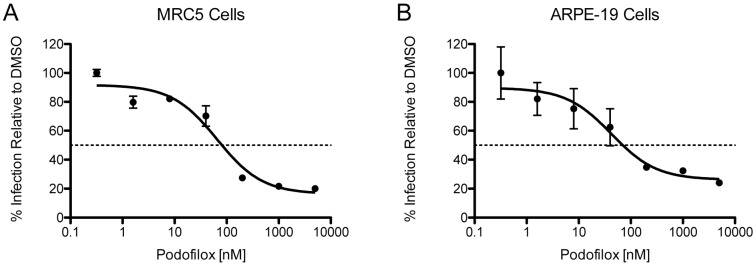
Podofilox efficacy is dependent on CMV strain and cell type. The fluorescent signal from TB40/E_WT_-infected MRC5 cells (**A**) and TB40/E_WT_-infected ARPE-19 cells (**B**) pretreated with varying doses of podofilox and stained at 18 h p.i. with murine anti-IE1 conjugated to Alexa fluorophore 488 was analyzed with an Acumen ^e^X3 cytometer. The number of virus-infected cells were used to determine percent infection with cells receiving DMSO pretreatment as 100% infection. Non-linear regression analysis was performed in Prism5. Points represent the mean of six replicates and error bars represent the standard error.

To determine if podofilox is effective against a clinical-like strain in multiple cell types, MRC5 or ARPE-19 cells were pre-treated with podofilox followed by infection with TB40/E_WT_. Virus infected cells were labeled with a murine anti-IE1 antibody conjugated with Alexa fluorophore 488 and analyzed at 18 h p.i. using an Acumen ^e^X3 cytometer to determine % infection. We observed a difference in the EC_50_ value of podofilox against TB40/E_WT_ (Figure 6 and Table 1) that could be due to TB40/E’s reliance on vimentin and intermediate filaments in contrast to AD169 [42]. What was most remarkable was the differences in the maximal plateau inhibition (MPI) [43] of podofilox based on Prism5’s non-linear regression (Figure 6 and Table 1). The MPI for TB40/E_WT_ in MRC5 cells is 84%; while in ARPE-19 cells, the MPI was reduced to 74%. These results may be due to the differences in viral entry of TB40/E in the different cell types. TB40/E contains the pentameric complex gH/gL/UL128/UL130/UL131a that allows the virus to enter via endocytosis followed by fusion with the endocytic membrane in a pH-dependent process in epithelial cells [44]. Yet, TB40/E entry in fibroblasts occurs by both cell surface fusion and endocytic fusion [17,44]. Thus, the ability of TB40/E to enter cells at the cell surface and a post-endocytosis step impacts the MPI of podofilox implying that podofilox is more effective at blocking virus entry when it inhibits entry at the cell surface.

### 3.4. Podofilox Inhibits Other Enveloped Viruses

**Figure 7:**
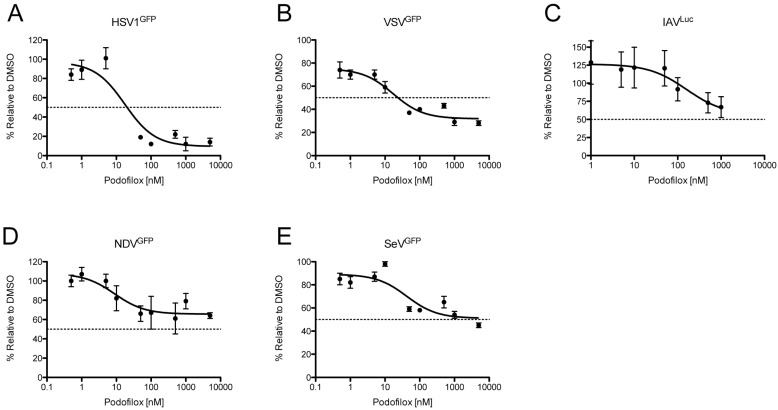
Podofilox inhibition of diverse enveloped viruses. MRC5 cells pretreated podofilox were infected with various reporter viruses and analyzed for GFP fluorescence (HSV1^GFP^, VSV^GFP^ NDV^GFP^, and SeV^GFP^) or luciferase activity (IAV^Luc^). The number of infected cells was determined using an Acumen ^e^X3 cytometer for HSV1^GFP^ (**A**, 24 h p.i.); VSV^GFP^ (**B**, 8 h p.i.); NDV^GFP^ (**D**, 24 h p.i.) and SeV^GFP^ (**E**, 24 h p.i.); ForIAV^Luc^ (**C**); the level of luciferase activity was quantified at 24 h p.i. on the BioTek Cytation^3^ imaging reader. The % infection was determined using DMSO-treated cells as 100% infection. Non-linear regression analysis was performed in Prism5. Points represent the mean of six replicates and error bars represent the standard error.

To determine if the antiviral effect of podofilox treatment was due to the viral mode of entry, the infection of diverse viruses [34,35] was examined in MRC5 cells pretreated with podofilox (Figure 7). MRC5 cells treated with increasing doses of podofilox (0–5 μM) were infected with diverse viruses that expressed GFP or luciferase and analyzed 8–24 h p.i. depending on the reporter virus. Based on EC_50_ values, podofilox treatment was extremely potent against HSV1^GFP^ and VSV^GFP^ (EC_50_ values: 20 and 24 nM, respectively) (Figure 7A,B and Table 2). However, analysis of MPI revealed that podofilox was a potent inhibitor for only HSV1^GFP^ with an MPI of 90% (Figure 7A and Table 2). Despite podofilox’s low EC_50_ for VSV^GFP^, the MPI for VSV^GFP^ was only 68% (Figure 7B and Table 2). Podofilox treatment was far less effective against IAV^Luc^, NDV^GFP^, and SeV^GFP^ with EC_50_ values >5 μM and MPI values of 44%, 34%, and 49%, respectively (Figure 7C–E and Table 2). The viruses tested enter cells by different mechanisms, and whether or not podofilox is an effective inhibitor may be dependent on the need for receptor clustering during virus entry. The viruses for which podofilox has low EC_50_ values and high MP—AD169 and HSV1—all engage receptors in a multi-step process prior to lipid fusion at the cell surface [15,45,46]; whereas IAV, NDV, and SeV—the viruses resistant to podofilox—engage putative sialic acid based receptors in a single step prior to lipid fusion [47,48,49]. Between those two phenotypes are TB40/E and VSV, viruses that engage receptors in a multistep process prior to lipid fusion in an endosome [20,50]. Collectively, podofilox inhibition of microtubules defines the requirement of the cytoskeleton network for virus to gain entry into cells.

### 3.5. Podofilox Does Not Disrupt Cellular Morphology

**Figure 8:**
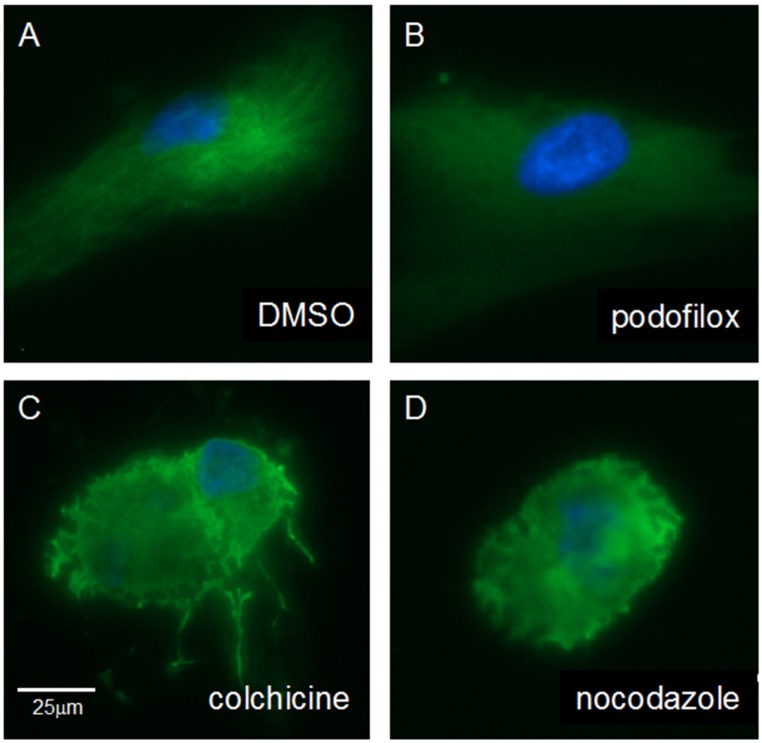
Fluorescent microscopy of MRC5 cells treated with microtubule inhibitors for 1 h. MRC5 cells were treated for 1 h with DMSO (**A**); 50 nM podofilox (**B**); 500 nM colchicine (**C**); or 5 μM nocodazole (**D**). Cells were stained with murine anti-tubulin antibody and visualized anti-mouse antibody conjugated to Alexa 488 (green) and nuclei stained with Hoescht reagent (blue). Scale bar is 25 μm.

MRC5 cells were treated for 1 h with DMSO, 50 nM podofilox, 500 nM colchicine, or 5 μM nocodazole for 1 h. Cells were stained for tubulin with anti-tubulin antibody followed by a visualized under the Evos FL cell imaging system (Figure 8). The nucleus was visualized using Hoescht reagent (Figure 8).

Intact microtubules can be visualized in DMSO treated cells (Figure 8A). Interestingly, the cells treated with podofilox demonstrated a diffuse microtubule with some appearance of tubule structures (Figure 8B). Yet, colchicine (Figure 8C) and nocodazole (Figure 8D) treatment caused disruption of the main microtubule networks of the cell as observed by cell rounding. Collectively, these images show that the low dose of podofilox capable of significantly inhibiting CMV infection does not drastically disrupt the microtubule systems of the cell at a time when virus would be entering the cells.

## 4. Discussion

The present study proposes a novel role for microtubules in CMV entry. The data demonstrates that podofilox inhibits an early step of virus entry. The collective results suggest that podofilox inhibits CMV entry following virus binding and stabilizes the virus in a pre-penetration step and immediately following virus penetration (Figure 2, Figure 3 and Figure 4). This is in contrast to other microtubule inhibitors, such as colchicine and nocodazole, that do not influence CMV penetration and are more likely during capsid transport to the nucleus. CMV entry into fibroblasts is a complex process involving different viral glycoproteins and associated complexes (gM/gN, gB, gH/gL/gL, and gH/gL/UL128/UL130/UL131a) involved in three distinct sequential phases (attachment, binding, and fusion) [46]. The fusion protein for CMV, gB, belongs to the newly discovered group of class III fusion proteins (which also contains HSV1 gB and VSV G) [51]. Little is known about the details of CMV entry, but for HSV1, gB’s membrane proximal region (MPR) is key for protecting the fusion loops (FLs) from prematurely interacting with the membrane, and that in the absence of that blockage, gB is able to better interact with the membrane as well as cluster together via lateral interactions with other gB molecules [52]. It is possible that gB binding to its receptor is the trigger for the release of the FLs by the MPRs and for allowing their clustering and enhancing virus entry. Given that there are many potential receptors for CMV gB, and several of them are cell signaling receptors that work in conjunction with other proteins [53,54,55,56,57], the interaction of numerous CMV gB molecules with multiple cellular proteins allowing gB to cluster and expand the fusion pore at the cell surface is critical for entry and is inhibited by podofilox. Since CMV, unlike other viruses, is able to enter via multiple entry routes in a strain dependent manner [19,20,58], the varied EC_50_ values and MPI between CMV strains and target cell type (Figure 6) further supports the role of microtubules at the plasma membrane for virus entry.

The anti-CMV properties of podofilox, a podophyllotoxin, are different from colchicine and nocodazole because of the differences in how these compounds interact with tubulin at the molecular level. Nocodazole treatment inhibited CMV infection at a post-entry step [23] implying that microtubules were not involved in the process of CMV entry. Their data are accurate, and were corroborated in this paper; however, we show that the anti-CMV properties of nocodazole are not applicable to all microtubule inhibitors and thus it appears that microtubules can be important for CMV entry dependent on the specific inhibitor. Colchicine binds to tubulin slowly and irreversibly, while podofilox and nocodazole both bind rapidly and reversibly [24]. While they are all capable of inhibiting polymerization, they have disparate effects on the monomers at the molecular level. For example, when colchicine is bound to tubulin, tubulin shows decreased GTPase activity and intrinsic fluorescence, but when podofilox is bound to tubulin, the opposite occurs [59]. Nocodazole, despite having a rapid, reversible binding similar to podofilox, also inhibits tubulin’s GTPase activity like colchicine [60]. Colchicine and nocodazole both induce conformational changes in tubulin, while podofilox does not [61]. The three ligands also differ in how they impact the effect of the tubulin degrading ligand IKP104 [24]. Additionally, during tubulin decay, sulfhydryl groups and hydrophobic areas of the tubulin monomers become exposed, and colchicine is more effective at inhibiting this process than podofilox [62,63]. This explains why podofilox is able to affect the more dynamic microtubules at the cell surface differently than the more stable intracellular microtubules—a phenomenon observed by other tubulin ligands [25].

Microtubules help regulate the localization of signaling complexes on the surfaces of cells that can be disrupted by microtubule depolymerizing agents [64]. Caveolin-1, in particular, has been implicated in controlling the membrane localization of proteins such as β1 integrins [65] that are important for CMV entry [15,16]. Additionally, β1 integrins interact with PDGFR, another cell surface receptor implicated in CMV entry [56], on the surface of lung fibroblasts [66]. It is therefore possible that the localization of these CMV entry factors, and thus the ability for CMV to complete its full binding and entry procedure, is altered in the presence of podofilox because of its effect on microtubules.

Podofilox’s antiviral effect on other viruses further supports the fact that it inhibits CMV entry by preventing a latter step of virus entry following initial virus binding. Podofilox potently inhibits infection by other viruses that also have multistep binding processes prior to infection. In contrast, viruses with single-step entry mechanisms are resistant to the effects of podofilox on the cortical microtubules. For IAV, NDV and SeV, single micromolar doses of podofilox (over 60× the EC_50_ of podofilox for AD169) was unable to inhibit infection at levels higher than >50%. The entry of these viruses is dependent on sialic-acid based receptors [47,48,49] that are extremely abundant on the cell surface and thus do not require as much microtubule mediated help to cluster together in sufficient density. The multistep entry process for the α-herpesvirus HSV1 [45] is similar to CMV, a β-herpesvirus and thus, the efficacy of podofilox is also very similar (EC_50_ of 20 nM vs. 26 nM [26], respectively). VSV also engages multiple receptors prior to entry [50] and has a low EC_50_ value of 24 nM. Yet, VSV is different from HSV1 by nature of entering the cell via pH dependent endocytosis instead of fusion at the cell surface, and is not fully inhibited by podofilox (MPI 68% vs. 90% for HSV1). The TB40/E strain of CMV is also capable of entering cells via endocytosis; however, the majority of the time it enters via cell surface fusion [17]. In MRC5 cells, the MPI of podofilox against TB40/E is 84%, very similar to the MPI of 88% for podofilox against AD169 due to the dependency of viral entry at the surface of fibroblasts. In ARPE-19 cells, however, the MPI is reduced to 74%. The remaining TB40/E (~26%) not inhibited by maximal podofilox treatment is a number similar to the estimated 35% of virus that enters ARPE-19s via endocytosis instead of cell surface fusion [17]. While the role of microtubules in post-fusion intracellular transport has been described for HSV1 [67] and VSV [68], 50 nM podofilox at one hour did not cause drastic effects on the general cytoplasmic microtubule machinery (Figure 8) and cannot account for a decrease in virus infection. Collectively, these findings propose a novel role for microtubule activity during the entry process of selective viruses, and the investigation of the exact role of how microtubules promote virus entry would lead to the establishment of a more comprehensive paradigm of virus infection.

## 5. Conclusions

In conclusion, this paper demonstrates the potential potency of microtubule inhibitors as CMV entry inhibitors and inhibitors of other viruses with multi-step entry processes involving cell surface fusion. Molecularly distinct microtubule inhibitors have such different effects on virus entry (Figure 2 and Figure 3) and on the whole cell’s microtubule network demonstrating that, theoretically, a potent, non-toxic inhibitor can be created that preferentially disrupts the microtubule networks necessary for CMV entry, and potentially other viruses, while sparing those required for normal cell function. Additionally, even lower doses of microtubule inhibitors from classes similar to the podophyllotoxins could be used in conjunction with other existing or novel CMV therapeutics, to mimic the cocktail therapy approach of HIV/AIDS therapy [69], and reduce the toxicity associated with each compound, while generating a greater antiviral effect.

## Figures and Tables

**Table 1:** EC_50_ and MPI values for podofilox’s inhibition of CMV infection.

Virus	Cell	EC_50_ (nM)	MPI (%)
TB40/E_WT_	MRC5	81	84
TB40/E_WT_	ARPE-19	70	74

**Table 2:** EC_50_ and MPI values for podofilox’s inhibition of infection by various viruses.

Virus	EC_50_ (nM)	MPI (%)
HSV1	20	90
IAV	N/A	44
NDV	N/A	34
SeV	N/A	49
VSV	24	68

## Abbreviations

The following abbreviations are used in this manuscript:

CMV	Cytomegalovirus
EC_50_	50% effective concentration
FL	Fusion loops
GFP	Green fluorescent protein
HSV1	Herpes simplex virus 1
IAV	Influenza A Virus
IE	Immediate early
LUC	Luciferase
MPI	Maximal plateau inhibition
MPR	Membrane proximal region
NDV	Newcastle disease virus
SeV	Sendai virus
UL	Unique long
US	Unique short
VSV	Vesicular stomatitis virus
YFP	Yellow fluorescent protein
